# Antenatal Care Service Utilization Among Adolescent Pregnant Women–Evidence From Swabhimaan Programme in India

**DOI:** 10.3389/fpubh.2019.00369

**Published:** 2019-12-12

**Authors:** Prakash H. Fulpagare, Abhishek Saraswat, Konsam Dinachandra, Nikita Surani, Rabi N. Parhi, Sourav Bhattacharjee, Somya S, Apollo Purty, Babita Mohapatra, Nita Kejrewal, Neeraj Agrawal, Vikas Bhatia, Manisha Ruikar, Raj Kumar Gope, Zivai Murira, Arjan De Wagt, Vani Sethi

**Affiliations:** ^1^International Institute for Population Sciences, Mumbai, India; ^2^Independent Consultant, New Delhi, India; ^3^Field Office, UNICEF, Patna, India; ^4^Field Office, UNICEF, Bhubaneswar, India; ^5^Bihar Rural Livelihoods Promotion Society, Patna, India; ^6^Odisha Livelihoods Mission, Bhubaneswar, India; ^7^Deendayal Antyodaya Yojana, National Rural Livelihoods Mission, New Delhi, India; ^8^All India Institute of Medical Sciences (AIIMS), Patna, India; ^9^All India Institute of Medical Sciences (AIIMS), Bhubaneswar, India; ^10^All India Institute of Medical Sciences (AIIMS), Raipur, India; ^11^Ekjut, Ranchi, India; ^12^Regional Office for South Asia, UNICEF, Kathmandu, Nepal; ^13^Country Office, UNICEF, New Delhi, India

**Keywords:** pregnant adolescent, pregnant women, teenage pregnancy, antenatal care, India

## Abstract

**Purpose:** Pregnant adolescent girls (15–19 years) are more vulnerable to poor health and nutrition than adult pregnant women because of marginalization and lack of knowledge about the antenatal care (ANC) services. The present study aims to test this hypothesis and assess determinants of ANC service utilization among currently adolescent pregnant women.

**Methods:** Data were drawn from the baseline survey of SWABHIMAAN project, which had been conducted in three states of India: Bihar, Chhattisgarh, and Odisha. Out of a total 2,573 pregnant women (15–49 years) included in the sample, about 10% (*N* = 278) were adolescent girls (15–19 years) at the time of the survey, and the rest were adults. Sample was selected from the population using simple random sampling, and information was collected using pretested questionnaires.

**Results:** For all indicators of ANC service utilization, performance of adolescent pregnant women was better than adult pregnant women. However, significant variations were reported in the level of services received by adult pregnant women for different indicators. Religion, wealth, food insecurity, Village Health Sanitation and Nutrition Day meeting, Public Distribution System and Integrated Child Development Services entitlements, and knowledge of family planning methods had a significant effect on the ANC service utilization.

**Conclusion:** Adolescent pregnant women have shown better utilization of selected indicators than their adult counterparts. Utilization of full ANC services starting from first trimester itself for adolescent pregnant women is an urgent need in present context. Intervention program must pay attention to such adolescent married girls who are entering into the motherhood phase of their lives.

## Introduction

India is home to 243 million adolescents (10–19 years) out of which 120 million are adolescent girls, accounting for nearly 10% of its population (1). Pregnancy in the adolescence period continues to be a challenge in low- and middle-income countries, with an estimated 16 million girls aged 15–19, and 2 million girls under the age of 15 years, becoming pregnant every year (2). World Health Organization (WHO) estimated that adolescent pregnancies account for 11% of all births globally (3). According to the National Family Health Survey-(NFHS)4, the prevalence of teenage pregnancy in India is 7.9% (4).

Pregnancy in adolescence is associated with adverse maternal and perinatal outcomes. Adolescent pregnancies account for nearly one-fourth of the total burden of disease due to obstetric factors among women, and around 95% of such births occurred in low- and middle-income countries, where pregnancy- and childbirth-related complications are the primary cause of fatality among adolescent girls (3). Teenage girls are biologically immature to handle pregnancy and are nutritionally vulnerable and thus have a risk of obstetric complications and adverse pregnancy outcomes (specifically, low birth weight; prematurity; small for gestational age births; and neonatal, postneonatal, and infant mortality) and morbidity (5, 6). A study on six low- to middle-income countries from sub-Saharan Africa, Latin America, and South Asia reported that adolescents are at significantly increased risk of delivering premature and low-birth-weight offspring, and the risk of neonatal and perinatal mortality is also higher among adolescents especially early adolescents (<15 years) (7). Children born to young mothers are disadvantaged in childhood nutrition and schooling (8).

The sociocultural and economical factors encompassing parental influence, unequal gender power relations, poverty, and low education are identified as significant determinants of adolescent pregnancy (9, 10). Additionally, factors like cost of contraceptives, accessibility to or availability of contraceptives, inhibition to use contraception, lack of comprehensive sex education, and misconceptions about contraceptives also affect adolescent pregnancies (11). Early marriage is one of the critical drivers of pregnancy and childbearing in adolescence, which are relatively common in many developing countries, including India (12, 13). In the South Asian region, one in five adolescent girls are currently married or in a union. In some parts of rural India, 27% of women aged 20–24 years are married before 18 years, and 8% give birth before completing the age of 18 years (4). A study from India revealed that close to 40% marriages that took place were child marriages, and nearly half of the pregnancies were teenage pregnancies (14).

The WHO guidelines suggest that pregnancy and adverse reproductive outcomes among adolescents can be avoided by generating awareness and preventing early marriages (15). The guidelines on improving adolescent health recommend increased utilization of skilled antenatal care (ANC), childbirth, and postnatal care among adolescents (16). Several studies (17, 18) have shown that most of the obstetric complications can be dodged by timely access to preventive and curative health services like ANC. ANC is essential for ensuring safe motherhood. The components of ANC include risk identification, prevention and management of pregnancy-related or concurrent diseases, nutritional problems, and health education and health promotion (19). Access to these services enables skilled health care professionals to identify and treat potential health risks to expecting woman and the unborn baby and ensures safe delivery (20).

In India, Reproductive and Child Health (RCH) Programme under the Ministry of Health and Family Welfare (MoHFW) advises to receive at least three ANC checkups, iron and folic acid (IFA) tablets, calcium tablets, tetanus toxoid (TT) injections, weight monitoring, blood pressure check, abdominal examination, and counseling by a frontline health worker (21). It is likely to happen that not all pregnant women who receive ANC services may utilize the entire package of services (full ANC). Some might register but never utilize, whereas some might utilize only part of the services, and others may not meet the minimum number of contacts required.

Several studies exploring factors associated with utilization and coverage of ANC services have been conducted (22, 23); however, these research efforts have focused on women of all reproductive age groups, with inadequate attention given to understanding factors associated with ANC utilization among adolescent pregnant women. According to the NFHS-4 survey, the proportion of ANC coverage among women aged 15–49 years has increased by 7% during 2005–2006 to 2015–2016 (4, 24). Around 59% of women of reproductive age received first ANC checkup during the first trimester of pregnancy, and 51% had more than three ANC visits (4), which is comparable to the global coverage of 58.6% and slightly higher than ANC coverage for South Asia (50.0%) (25). There are notable variations in the exploitation of ANC services by younger (adolescents) and older (adult) pregnant women. Adult pregnant women are more likely to utilize ANC as compared to adolescent pregnant women, and a number of socioeconomic and demographic factors like education, employment, income, place of residence, geographical variations, birth order, and parity explain the differences in utilization of ANC services among adolescent and adult pregnant women (26–28). However, there is insufficient evidence available exclusively on the use of ANC services by adolescent pregnant women, and their specific vulnerabilities (17, 29, 30). Exploring the ANC utilization among adolescent pregnant women and adult pregnant women may be useful to spot disparities, distinguish barriers, and suggest appropriate measures to enhance the uptake of ANC services.

Therefore, this study aims to (i) analyze and compare the exploitation of ANC services by currently pregnant adolescent women and adult pregnant women and (ii) explore the determinants of ANC service utilization among adolescent pregnant women.

## Materials and Methods

### Study Area and Population

**Figure 1:**
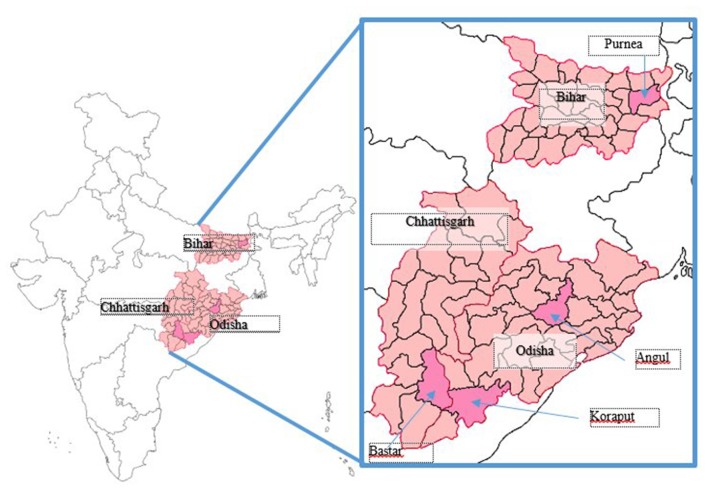
Coverage area of the Swabhimaan programme.

Data for this study are based on the baseline household survey of SWABHIMAAN intervention program conducted in 2016. The coverage area of the survey was three states located in the eastern region of India, namely, Bihar, Chhattisgarh, and Odisha (Figure 1). These states share 15% of scheduled castes (SC)/scheduled tribes (ST) population. Combined, more than one-fourth of the total population in the region is SC/ST, and around 85% of the people reside in rural areas. The region is poverty-stricken with over one-third of the population is living below the poverty line (BPL) (31). The fourth round of the NFHS-4 recorded that around 44% women in Bihar, 4% in Chhattisgarh, and 6% of women in Odisha did not get any ANC during the pregnancy for their latest live birth. Only 3, 22, and 23% of women in Bihar, Chhattisgarh, and Odisha, respectively, received full ANC (at least four ANC visits, 1 TT injection, and taken IFA for at least 100 days) during the pregnancy for their last live birth. NFHS-4 also showed that a high proportion of adolescent girls (15–19 years) in this region have a low body mass index (BMI) (over 37%) (4). The study was part of a prospective, nonrandomized, controlled evaluation registered under the Clinical Trials Registry-India (CTRI) (32). SWABHIMAAN is a multistate, multisector women's nutrition demonstration intervention program with an endeavor to improve the nutrition of women. The baseline survey data collection in all three states was conducted between July and December 2016. The baseline survey collected data from currently pregnant women (adolescent pregnant women and adult pregnant women). “Pregnant women” were defined as all pregnant women of age 15–49 years, whether they were an adolescent or adult. All the participants were currently pregnant at the time of the survey.

### Study Procedures

A pretested questionnaire was prepared to obtain information on the sociodemographic profile, household characteristics, and diet diversity. The questionnaire also covered information on access to health and nutrition services like the Integrated Child Development Services (ICDS) and the State Rural Livelihood Mission (named as JEEViKA in Bihar, AJEEViKA in Chhattisgarh, and the Odisha Livelihood Mission in Odisha), aimed at social and economic empowerment of rural poor, ANC services utilization, knowledge of family planning, and decision-making power. The bilingual questionnaires had questions in both English and the local languages of the respective states. The investigators for data collection were given 1 week of intensive training. Seven supervisors supervised the data collection teams. The questionnaires were administered to pregnant women through face-to-face computer-assisted personal interviewing (CAPI) by the investigators. Control checks were done for 10% of the sampled women to maintain the quality of the data.

### Sample Size

The Swabhimaan baseline sample was designed to provide estimates of key indicators at district levels (for one district in Bihar, two districts in Odisha, and one district in Chhattisgarh). The total sample size of 2,571 households for pregnant women was based on the size needed to produce reliable indicator estimates for each district and selected blocks in selected districts. The study was conducted in rural settings. The sample was selected through a two-stage sampling design. In the first stage, PSUs were selected where villages served as the primary sampling units (PSUs). In the second stage, a complete mapping operation was conducted to list the households in the selected PSUs, and then the households were selected through random sampling. From each household, one pregnant woman was randomly selected.

In Bihar, Kasba, and Jalalgarh, blocks of Purnea district were divided into three clusters of 27, 41, and 36 villages, and where cluster 2 and cluster 3 served as intervention and control areas, respectively. In Bastar district, Chhattisgarh, 40 villages each were selected from Bastar block and Bakawand block, where former served as intervention area and the latter as control area. In Odisha, survey was done in Koraput and Pallahara districts, from each of the districts, six intensive and seven non-intensive village Panchayats were selected to serve as intervention and control areas, respectively. Therefore, based on the outcome indicators and the change envisaged, the representative sample of 936 (Bihar), 823 (Chhattisgarh), and 814 (Odisha) pregnant women were selected from the three states. Thus, a total sample of 2,571 pregnant women was derived. Out of the total sample size of 2,571 pregnant women, more than 10% (*n* = 278) adolescents were pregnant (Bihar: 75, Chhattisgarh: 75, and Odisha: 128).

### Ethical Clearance

Ethical approval was taken from the Institutional Ethics Committee of All India Institute of Medical Sciences (AIIMS) of the respective states, namely, Bihar, Chhattisgarh, and Odisha. The impact evaluation was registered with the Registry for International Development Impact Evaluations (RIDIE-STUDY-ID-58261b2f46876) and the National Clinical Trials Registry of India with trial number CTRI/2016/11/007482 (33). Verbal consent was obtained from interviewed pregnant women. In the case of adolescent respondents, verbal consent was from the respondents as well as their parents/guardians. All India Institute of Medical Sciences Bihar, Chhattisgarh, and Odisha played a key role in data collection and compilation and hiring and imparting intensive training to the field investigators, with technical support of the International Institute for Population Sciences and UNICEF.

### Significance of Data

In many large-scale surveys (e.g., NFHS, DLHS), the information about ANC services availed during the latest pregnancy is collected retrospectively based on recall of events from the last pregnancy. Whereas, in the SWABHIMAAN baseline survey, the detailed information regarding ANC registration, utilization of ANC services, dietary practices, and decision-making power was asked to the currently pregnant women who are one of the target groups of the SWABHIMAAN program. Therefore, these data are unique and one of its kind as far as studying the utilization of ANC services of pregnant women is concerned as it minimizes misreporting due to recall bias.

### Data Analysis

The survey data from currently pregnant women (adolescent pregnant women and adult pregnant women) were used to assess factors related to the utilization of ANC services among currently pregnant adolescent girls and adult pregnant women and the determinants of ANC service utilization among adolescent pregnant women. The software STATA 14.0 was used for data analysis. The dependent variable included utilization of ANC services, which included registration of pregnancy, weight monitoring, counseling from frontline health workers, TT injection, IFA tablets, deworming, and calcium tablets, whereas the independent variables included household characteristics and sociodemographic characteristics of the adolescent pregnant women and adult pregnant women. Descriptive statistics were used to study the characteristics of the sample. Chi-square statistics were used to report differences in ANC utilization among pregnant adolescents and adult pregnant women by category of services at a 95% level of significance. The logistic regression analysis was done separately to assess the associations of each component of ANC with the household characteristics, socioeconomic support, and knowledge and decision-making power at the 95% level of significance. Regression model had significant χ^2^ value at *p* < 0.001 at 95%. Results of the regression analyses are presented in the form of odds ratios (OR), with 95% confidence intervals (CI) and *p*-values.

## Results

### Sociodemographic Characteristics of Currently Pregnant Women

The analysis population included 2,571 currently pregnant women (Table 1). The mean age of the sample was 24.9 years (pregnant adolescents 18.1 years and pregnant adult women 25.8 years). Among all of the pregnant women, nearly 11% were adolescents; the majority of pregnant women were in the 20–24 years old age group (41.9%) followed by the 25–29 years age group (31.0%). Only 5.5% of pregnant women were above the age of 34 years. Twenty-seven percent of the adolescent pregnant women were illiterate as compared to 47% of the adult pregnant women. Education status of adolescent girls was better than adult pregnant women. Over 50% of adolescent pregnant women had 1–9 years of education, and another 20% had an education of 10 years and more; however, only 40 and 13% adult pregnant women had the former and latter education levels, respectively. Three out of four pregnant women were nonworking. Overall, nearly 16% of pregnant women were in the first trimester of pregnancy, 37% in the second trimester, and 47% of women were in the third trimester of pregnancy. Slightly over 70% of women had a parity of two or more. About 79% of the sampled pregnant women practiced the Hindu religion, and the majority of women belonged to scheduled tribes (41%) and other backward classes (39%). Based on wealth, 34% of pregnant women belonged to low wealth tertile, and 33% of women belonged to the middle and high wealth tertile each. Furthermore, about 25% of adolescent pregnant women and 39% of adult pregnant women had BPL cards, which are issued by the government for availing subsidized ration. Only 7% of pregnant adolescent women were members of Self-Help Groups (SHGs).

**Table 1:** Sociodemographic characteristics of currently pregnant women in Bihar, Chhattisgarh, and Odisha.

**Characteristics**	**Adolescent pregnant women (*N* = 278) % (*n*)**	**Adult pregnant women aged 20–49 years (*N* = 2,293) % (*n*)**	**Total (*N* = 2,571) % (*n*)**
**Mean age (years)**	18.1	25.8	24.9 (2,571)
**Age group (years)**			
15–19	100.0 (278)	–	10.8 (278)
20–24	–	46.9 (1,076)	41.9 (1,076)
25–29	–	34.8 (798)	31.0 (798)
30–34	–	12.2 (279)	10.8 (279)
35–39	–	4.6 (105)	4.1 (105)
Above 40	–	1.5 (35)	1.4 (35)
**Education**			
Non-literate	27.0 (75)	47.2 (1,081)	45.0 (1,156)
1–9 years	52.9 (147)	39.8 (912)	41.2 (1,059)
10 years and more	20.2 (56)	13.1 (300)	13.9 (356)
**Working**			
No	82.1 (228)	73.9 (1,693)	74.8 (1,921)
Yes	18.0 (50)	26.2 (600)	25.3 (650)
**Trimester of pregnancy**			
First	20.1 (56)	15.4 (352)	15.9 (408)
Second	41.4 (115)	37.0 (849)	37.5 (964)
Third	38.5 (107)	47.6 (1,092)	46.6 (1,199)
**Parity**			
2+	22.3 (62)	77.1 (1,768)	71.2 (1,830)
1	77.7 (216)	22.9 (525)	28.9 (741)
**Religion**			
Hindu	86.7 (241)	77.6 (1,779)	78.6 (2020)
Others	13.4 (37)	22.5 (514)	21.5 (551)
**Caste**			
Scheduled caste	10.8 (30)	13.4 (307)	13.2 (337)
Scheduled tribe	43.6 (121)	40.6 (931)	41.0 (1052)
Other backward classes	35.7 (99)	38.9 (891)	38.6 (990)
Others	10.1 (28)	07.2 (164)	07.5 (192)
**Wealth status**			
Low	38.9 (108)	33.6 (769)	34.2 (877)
Middle	33.1 (92)	32.5 (745)	32.6 (837)
High	28.1 (78)	34.0 (779)	33.4 (857)
**Have BPL card**			
No	74.8 (208)	61.1 (1,401)	62.6 (1,609)
Yes	25.2 (70)	38.9 (892)	37.4 (962)
**SHG membership**			
No	92.9 (258)	73.2 (1,677)	75.3 (1,935)
Yes	07.2 (20)	26.9 (616)	24.8 (636)

### ANC Services Utilized by Currently Pregnant Women

Differences in utilization of various ANC services between the pregnant adolescent and adult pregnant women groups are shown in Table 2. A significantly higher proportion of pregnant adolescent women (83.9%) registered the pregnancy as compared to adult pregnant women (78.6%, *p* < 0.05, CI 95%). Around 63% of pregnant adolescents registered the pregnancy in the first trimester, which is a considerably higher proportion compared to only 51% of adult pregnant women (*p* < 0.01). There was not much difference in the level of weight monitoring during pregnancy among pregnant adolescents (70.2%) and adult pregnant women (68.5%). Among pregnant adolescents, 55.4% received counseling from frontline health workers, whereas only 50.9% of adult pregnant women received counseling. A slightly less proportion of adult pregnant women got tetanus injection (69.0%) and deworming tablets (9.1%) in the second and third trimesters of pregnancy as opposed to pregnant adolescents (72.7 and 12.0%, respectively). A significantly higher proportion of pregnant adolescents (73.3%, *p* < 0.05) received IFA tablets during pregnancy compared to adult pregnant women (66.5%). Similarly, a significantly lesser proportion of adult pregnant (20.7%) women received calcium tablets during the second and third trimesters of pregnancy than pregnant adolescents (30.9%, *p* < 0.01).

**Table 2:** Antenatal care (ANC) service utilization among currently adolescent pregnant women and adult pregnant women in sampled households.

**ANC services**	**Adolescent pregnant women (*N* = 278) *n* (%)**	**Pregnant women (20–49 years) (*N* = 2,293) *n* (%)**	**Total (*N* = 2,571) *n* (%)**	**χ^**2**^**	***p*-value**
Pregnancy registered	233 (83.9)	1,802 (78.6)	2,035 (79.2)	4.1	0.043
Registered in first trimester	176 (63.4)	1,178 (51.4)	1,354 (52.7)	14.2	0.000
Weight monitored during pregnancy	195 (70.2)	1,569 (68.5)	1,764 (68.7)	0.3	0.560
Counseling from frontline health workers	154 (55.4)	1,165 (50.9)	1,319 (51.3)	2.1	0.148
Tetanus Injection (second and third trimester)	202 (72.7)	1,582 (69.0)	1,784 (69.4)	1.6	0.210
IFA received (second and third trimester)	159 (73.3)	1,203 (66.5)	1,362 (67.3)	4.0	0.045
Deworming taken (second and third trimester)	26 (12.0)	163 (9.1)	189 (9.4)	2.0	0.155
Calcium received (second and third trimester)	67 (30.9)	373 (20.7)	440 (21.8)	12.0	0.001

### ANC Utilization Among Pregnant Women by Background Characteristics

Table 3 shows the utilization of different ANC services by pregnant adolescent women according to background characteristics.

**Table 3:** Proportion of adolescent pregnant women with access to antenatal care services during pregnancy by personal, households, and socioeconomic background characteristics in sampled households.

	**All pregnant adolescent women**				**Adolescent pregnant women in second and third trimesters**				
	**Pregnancy registered in first trimester**	**Weight monitored in pregnancy**	**Counseling from frontline health worker**	**Total (*N*)**	**Received tetanus injection**	**Received iron and folic acid tablet**	**Took deworming tablet**	**Received calcium tablet**	**Total (*N*)**
**PERSONAL CHARACTERISTICS**									
**Education attainment**									
Non-literate	57.3	70.7	64.0	75	93.4	70.5	13.1	24.6	61
1–9 years	61.9	66.0	51.0	147	91.0	71.2	10.8	28.8	111
10 years and more	75.0	80.4	55.4	56	97.8	82.2	13.3	44.4	45
**Parity**									
2+	62.9	62.9	58.1	62	87.8	67.4	18.4	26.5	49
1	63.4	72.2	54.6	216	94.6	75.0	10.1	32.1	168
**Working**									
No	61.8	68.9	54.8	228	93.3	73.0	13.5	32.6	178
Yes	70.0	76.0	58.0	50	92.3	74.4	5.1	23.1	39
**HOUSEHOLD CHARACTERISTICS**									
**Religion**									
Hindu	67.2	74.7	58.1	241	94.7	78.7	12.8	33.0	188
Other religion	37.8	40.5	37.8	37	82.8	37.9	6.9	17.2	29
**Caste**									
Scheduled caste	56.7	73.3	50.0	30	95.8	66.7	20.8	41.7	24
Scheduled tribe	71.1	70.3	55.4	121	92.6	81.1	6.3	27.4	95
Other backward classes	56.6	66.7	55.6	99	92.2	68.8	16.9	31.2	77
Other	60.7	78.6	60.7	28	95.2	61.9	9.5	33.3	21
**Households having ration card**									
No	57.7	71.2	50.0	104	92.7	68.3	7.3	26.8	82
Yes	66.7	69.5	58.6	174	93.3	76.3	14.8	33.3	135
**Wealth status**									
Low	63.0	73.2	56.5	108	91.9	74.4	12.8	29.1	86
Middle	58.7	59.8	54.4	92	91.7	68.1	11.1	31.9	72
High	69.2	78.2	55.1	78	96.6	78.0	11.9	32.2	59
**Food insecurity scale**									
Food secure	63.3	70.6	55.1	109	92.8	75.9	7.2	31.3	83
Mild	58.3	68.8	58.3	48	92.1	68.4	10.5	23.7	38
Moderate	61.8	68.5	52.8	89	97.1	72.1	16.2	35.3	68
Severe	75.0	75.0	59.4	32	85.7	75.0	17.9	28.6	28
**Dietary diversity scores**									
Less than five	62.4	72.2	48.9	133	92.5	68.9	8.5	25.5	106
Five or above	64.1	68.3	61.4	145	93.7	77.5	15.3	36.0	111
**SOCIAL AND ECONOMIC SUPPORT**									
**SHG membership**									
No	62.8	71.3	54.7	258	93.5	73.1	11.9	31.8	201
Yes	70.0	55.0	65.0	20	87.5	75.0	12.5	18.8	16
**Attended VHSND meeting**									
No	55.2	61.5	44.3	174	91.2	64.8	7.2	24	125
Yes	76.9	84.6	74.0	104	95.7	84.8	18.5	40.2	92
**Get ration from PDS**									
No	56.5	69.6	53.6	138	92.7	69.7	14.7	33	109
Yes	70	70.7	57.1	140	93.5	76.9	9.3	28.7	108
**Received double amount of ICDS food**									
No	56.3	62.3	43.1	167	91.9	65.3	9.7	22.6	124
Yes	73.9	82.0	73.9	111	94.6	83.9	15.1	41.9	93
**KNOWLEDGE AND DECISION-MAKING POWER**									
**Knowledge of family planning**									
No	62.6	71.8	54.4	206	91.6	73.1	12.6	31.7	167
Yes	65.3	65.3	58.3	72	98.0	74.0	10.0	28.0	50
**Decision about own health care**									
No	60.6	69.7	55.1	109	94.1	76.2	9.5	33.3	84
Yes	65.1	70.4	55.6	169	92.5	71.4	13.5	29.3	133
**Decision about major household purchase**									
No	58.7	68.8	49.5	109	91.9	70.9	4.7	29.1	86
Yes	66.3	71.0	59.2	169	93.9	74.8	16.8	32.1	131
**Decision about visiting relative**									
No	47.1	69.6	46.1	102	94.9	65.4	6.4	24.4	78
Yes	72.7	70.5	60.8	176	92.1	77.7	15.1	34.5	139
Total	63.3	70.1	55.4	278	93.1	73.3	12	30.9	217

#### Pregnancy Registration in the First Trimester

Sixty-three percent of the adolescents registered their pregnancy in the first trimester as shown in Table 3. Pregnancy registration in the first trimester increased with the increasing educational attainment of adolescents. The registration in the first trimester was highest among working adolescents and those from high wealth status. Based on the household characteristics, registration in the first trimester was highest among Hindu (67%) and Tribal (71%) adolescents. Registration in the first trimester was higher in pregnant adolescents from households possessing ration cards (67%) and having severe food security (75%). Also, pregnancy registration in the first trimester was highest among pregnant adolescents who received some form of socioeconomic entitlement support [self help group (SHG), public distribution system (PDS), village health sanitation and nutrition day (VHSND), and integrated child development services (ICDS)], knew about family planning, and had decision-making power.

#### Weight Monitoring During Pregnancy

Around 70% of pregnant adolescent women had their weight monitored during pregnancy (Table 3). Weight monitoring was highest among adolescents who were educated (10 or more years of education), working, and Hindu and those who belonged to other caste groups and high wealth status. In adolescent pregnant women, weight monitoring was highest among those who were food insecure (75.0%) and had low diet diversity (72%). Majority of pregnant adolescents who attended VHSND meetings and received PDS ration and double ICDS food had their weight monitored during pregnancy. Weight monitoring during pregnancy was highest among pregnant adolescent women having family planning knowledge and decision-making power (65–71%).

#### Availing Counseling From a Frontline Health Worker

More than half of the adolescent pregnant women received counseling from a health worker (55.4%). A higher proportion of pregnant adolescents who were illiterate (64%) received counseling as compared to educated pregnant adolescents (51–55%). The highest proportion of pregnant adolescents who were Hindu, belonged to other castes, had a diverse diet, availed socioeconomic support services (SHG, PDS, ICDS, VHSND), had knowledge of family planning and had decision-making power received counseling from frontline health worker during pregnancy.

#### ANC Services Received During the Second and Third Trimesters

In the second and third trimesters of pregnancy irrespective of the background characteristics, more than 90% of the adolescent pregnant women received tetanus injections, except for adolescents who were non-uniparous, non-Hindu, severely food insecure, and SHG members (Table 3). Seventy-three percent of adolescent pregnant women received IFA tablets in the last two trimesters. A higher proportion of adolescent women who received IFA tablets were educated, working, Hindu, tribal, and severely food insecure. During the second and third trimesters of pregnancy, only 12 and 31% of pregnant adolescents received deworming and calcium tablets, respectively. Utilization of various ANC services was higher among pregnant adolescents who utilized health and nutrition and socioeconomic support services (SHG, PDS, VHSND, ICDS), who knew about family planning and who had decision-making power regarding self-health care, major household purchases, and visiting relatives.

### Determinants of ANC Service Utilization

Table 4 shows the associations between ANC services utilization among adolescent pregnant women and different covariates, which include geographic, household, personal characteristics, social and economic support, knowledge, and decision-making power.

**Table 4:** Odds of ANC services utilization among adolescent pregnant women in sampled households.

	**Adolescent pregnant women**			**Adolescent pregnant women in second and third trimesters**			
**Variables**	**Pregnancy registered in first trimester**	**Weight monitored during pregnancy**	**Counseling from frontline health workers**	**Received tetanus injection**	**Received iron and folic acid tablet**	**Taken deworming tablet^**∧**^**	**Received calcium tablet**
**State**							
Bihar^®^							
Chhattisgarh	1.33	0.17^***^	0.55	0.02^**^	0.49		0.05^**^
Odisha	5.42^***^	0.24^**^	1.93	0.02^**^	0.91		5.26^**^
**PERSONAL CHARACTERISTICS**							
**Education attainment**							
Nonliterate^®^							
1–9 years	2.10^**^	1.08	0.58	0.77	1.13	1.15	2.57^**^
10 years and more	2.29^*^	1.93	0.44^*^	2.87	1.84	1.35	3.71^**^
**Parity**							
2+^®^							
1	1.28	1.63	1.19	2.81	1.09	0.54	1.95
**Work**							
No^®^							
Yes	1.17	2.21^*^	1.03	2.05	1.13	0.36	2.28
**HOUSEHOLD CHARACTERISTICS**							
**Religion**							
Hindu^®^							
Other religion	0.81	0.1^***^	0.82	0.02^**^	0.14^***^	0.39	0.64
**Caste**							
Scheduled caste^®^							
Scheduled tribe	2.04	0.64	1.38	0.46	2.25	0.48	0.47
Other backward classes	2.14	0.74	2.8^**^	0.38	1.98	3.21	1.13
Other	1.84	0.84	2.39	0.18	0.36	0.47	0.66
**Households having ration card**							
No^®^							
Yes	0.65	0.80	1.04	1.33	0.78	3.66^*^	1.88
**Wealth status**							
Low^®^							
Middle	1.12	0.54^*^	1.34	0.80	1.20	1.31	1.14
High	1.37	1.35	0.77	2.17	1.25	0.64	0.56
**Food insecurity scale**							
Food secure^®^							
Mild	0.64	0.61	0.91	0.62	0.48	1.58	0.62
Moderate	1.06	0.77	0.44^**^	2.12	0.95	5.44^**^	1.07
Severe	2.23	0.97	0.66	0.94	1.59	6.47^**^	0.39
**Dietary diversity scores**							
Less than five^®^							
Five or above	0.79	0.57^*^	1.81^**^	0.87	1.42	2.43	1.43
**SOCIAL AND ECONOMIC SUPPORT**							
**SHG membership**							
No^®^							
Yes	2.24	0.40	2.00	0.23	1.96	0.89	0.28
**Attended VHSND meeting**							
No^®^							
Yes	1.20	4.60^***^	2.68^***^	3.49	1.50	2.97^*^	1.68
**Get ration from PDS**							
No^®^							
Yes	2.37^**^	1.29	1.40	0.86	1.19	0.29^**^	0.81
**Received double amount of ICDS food**							
No^®^							
Yes	1.58	3.11^***^	4.00^***^	2.65	3.19^***^	1.12	2.62^**^
**KNOWLEDGE AND DECISION MAKING POWER**							
**Knowledge of family planning**							
No^®^							
Yes	1.25	0.55^*^	1.49	3.18	0.82	0.62	0.88
**Decision about own health care**							
No^®^							
Yes	0.58	1.35	0.65	1.00	0.32^**^	0.56	0.70
**Decision about major household purchase**							
No^®^							
Yes	1.04	1.46	1.56	5.67^*^	2.30	4.36^*^	1.15
**Decision about visiting relative**							
No^®^							
Yes	2.33^*^	0.41^*^	1.26	0.13	1.02	1.16	0.63
Constant	0.11^***^	9.75^**^	0.24^*^	513.2^**^	1.46	0.01^***^	0.06^***^

® *Reference category*,

* *p < 0.10*,

** p < 0.05, and

*** *p < 0.01 level of significance and ^∧^State not included in the model*.

Adolescent pregnant women from Odisha were five times more likely to register their pregnancy in the first trimester (*p* < 0.01) as compared to adolescents in Bihar. Pregnant adolescents having 10 or more years of education were more likely to register their pregnancy in the first trimester as compared to illiterates (*p* < 0.10). Adolescent pregnant women receiving ration from PDS (*p* < 0.05) and having the decision-making power to visit relatives (*p* < 0.10) were two times more likely to register pregnancy in the first trimester. Adolescent pregnant women from Odisha and Chhattisgarh were significantly less likely to have their weight monitored during pregnancy as compared to their counterparts from Bihar (*p* < 0.01 and *p* < 0.05, respectively). Working pregnant adolescent women were two times more likely to have their weight monitored during pregnancy (*p* < 0.10). Non-Hindu adolescent women were 90% less likely to have their weight monitored during pregnancy as compared to Hindus (*p* < 0.01). Middle-class adolescents and those having diet diversity were less likely to get weighed (*p* < 0.10). Pregnant adolescents receiving double food from ICDS and attending VHSND meeting were three to four times more likely to get weighed as compared to those who did not (*p* < 0.01). Adolescents having family planning knowledge and decision-making power to visit relatives were less likely to have their weight monitored during pregnancy (*p* < 0.10). More educated pregnant adolescents were less likely to receive counseling from frontline health workers during pregnancy (*p* < 0.10). Among pregnant adolescent women, diet diversity (*p* < 0.05), participation in VHSND meeting, and double food from ICDS (*p* < 0.01) were positively associated with receiving counseling from frontline health workers.

Among adolescent pregnant women in their second and third trimesters, those from Odisha and Chhattisgarh had significantly lower odds of receiving tetanus injection (*p* < 0.05) as compared to their counterparts from Bihar (Table 4). As compared to Hindu, non-Hindu adolescent pregnant women were less likely to receive tetanus injection (*p* < 0.05). Pregnant adolescents who could take decisions regarding major household purchases were five times more likely to receive tetanus injection (*p* < 0.10). Among pregnant adolescents, getting double food from ICDS was positively associated with receiving IFA tablets during pregnancy (*p* < 0.01). Pregnant adolescents who attended VHSND meeting, possessed ration card, and took decisions about major household purchases were three to four times more likely to receive deworming tablets (*p* < 0.10). Adolescent women facing moderate or severe food insecurity were four to six times more likely to receive deworming tablets during the second and third trimesters of pregnancy (*p* < 0.05). As compared to pregnant adolescents in Bihar, those in Chhattisgarh were less likely to receive calcium tablets, and on the other hand, pregnant adolescents in Odisha were five times more likely to receive calcium tablets (*p* < 0.05). Among pregnant adolescents, education and getting double ICDS food were positively associated with receiving calcium tablets (*p* < 0.05).

## Discussion

We evaluated the factors associated with utilization of ANC services among currently adolescent pregnant women (15–19 years) and adult pregnant women (20–49 years) and further explored the determinants of ANC service utilization among currently pregnant adolescent women. This study showed that around 8 in 10 pregnant women registered their pregnancy, but only half of the women registered the pregnancy in the first trimester and received counseling from frontline health worker. In the case of ANC services utilization in the second and third trimesters, a little over two-thirds of women received tetanus injection and IFA tablets. Only 1 in 10 women took the deworming tablet, and 2 in 10 received calcium tablets. These results reinforce the findings of work done in other parts or states of India like tribal areas of Maharashtra (34) and rural parts of Assam (35) where the registration of pregnancy was high (80%), but registration in the first trimester was 50–60%, whereas others (36) reported low registration rates varying from 8% in Madhya Pradesh and Rajasthan to 13–18% in Chhattisgarh and Odisha. It was observed that ANC utilization among pregnant adolescents was significantly better than adult pregnant women. However, other studies have reported that there is no difference between adolescents and adult pregnant women regarding the utilization of ANC services (26, 37). Our study demonstrated that education, wealth, utilization of socioeconomic support services (PDS, SHG, ICDS, and VHSND), knowledge of family planning, and decision-making power of women are significantly associated with utilization of ANC services and favor pregnant adolescents.

### Strengths and Limitations

It is important to mind the strengths and limitations of this study. The study has a robust design and contributes to a growing literature on factors that influence the utilization of ANC services among pregnant adolescent women and adult pregnant women. The findings could be used for planning for effective prenatal care. Most of the research on ANC utilization is based on collecting pregnancy-related data retrospectively. On the contrary, what makes this study more plausible is that the sample consisted of currently pregnant women, which minimized the recall period and chances of recall bias. Interviewing women who were currently pregnant ensured that women remembered details about ANC visits and whether they accessed any services offered as part of ANC. Another plus point of the study is the unique finding showing the higher utilization of ANC by adolescents in the study area. All interviewed currently pregnant women were at different stages of their pregnancy. Thus, each pregnant would give different information about the services she received depending upon the stage of pregnancy which might influence the statistics. The fact that the stage of pregnancy of respondents was not considered could be a limitation of the study. An important area that our study was unable to capture is the issue of service availability, accessibility, and satisfaction, which determine service utilization to a great extent.

Few critical inferences can be drawn from the results with implications on policy and program design to make maternal programs more inclusive and responsive to adolescent pregnant women's needs. First, it has been documented that pregnancy registration is one of the first and crucial steps to introduce pregnant women to maternal health services and provides a possibility to stimulate pregnant adolescents toward the use and importance of ANC services and its long-term health benefits for the young mothers and the offspring (38–40). Studies have shown that adolescent pregnant women are more likely to not avail or get late or infrequent ANC checkups compared to adult pregnant women (29, 41–44). On the contrary, our study reported that registration of pregnancy and enrollment in the first trimester was higher among adolescent pregnant women compared to adult pregnant women. However, pregnancy registration was lower among adolescents who were uneducated, nonworking, poor, and lacked participation in socioeconomic support programs. Several factors explain the disparities in the use and timing of ANC service utilization, such as lack of awareness, sociodemographic factors, personal traits (45), individual perceptions, and distance from the health facility (46). Hence, along with improvement in the socioeconomic status of women, it is also essential to focus on information, education, and communication (IEC) activities on educating women and their families about the importance of pregnancy registration in the first trimester. Thus, strenuous efforts are required to increase the accessibility to maternal health care services and improve the quality of services for those who do avail services (46). Also, pregnant mothers should be encouraged for early registration, which helps detect any abnormalities and take appropriate and timely actions (47).

Second, a significantly higher proportion of adolescent pregnant women received IFA, calcium and deworming tablets, weight monitoring, and counseling from health worker as compared to adult pregnant women, which shows the progress India has made regarding expanding the reach of maternal health care services to the areas that need them the most. The Rashtriya Kishor Swasthya Karyakram, a program strategized in 2014 by the Government of India, considers sexual and reproductive health of adolescents as an important component, including readiness for birth (48). However, the substantially low uptake of calcium tablets and deworming tablets indicated the need to sensitize individual and community toward full ANC.

Third, regarding the statewise comparison, this study showed that Odisha had significantly higher first trimester registrations compared to Bihar. An in-depth analysis of the NFHS-4 data to identify high priority districts of rural Bihar showed that only about a third of the pregnant women had ANC checkup in the first trimester, 13% received four ANC visits, and only 9% consumed IFA tablets for 100 days (4). The district identified for our study, Purnea, had one of the lowest literacy rates in Bihar. Receiving four ANC visits was lowest in Purnea district (7.7%) among all the identified districts. Purnea also had the highest proportion of anemic women—over 70% (49). It is interesting to note that apart from sociodemographic factors, motivation by family members and lower parity are found to be positively associated with complete ANC utilization among tribal women in Odisha, whereas in Chhattisgarh, the amount of time devoted by health staff for ANC checkup affected complete utilization of ANC services (45).

Fourth, sociodemographic factors, such as religion, caste, education, work status, income levels, and food security are significant determinants of ANC utilization among pregnant adolescents. Social and economic support through PDS and ICDS emerged as strong factors that can be used to strengthen programs for ANC. Our study showed that a higher number of adolescents who attend VHSND meetings had access to ICDS services and availed weight monitoring and counseling services. The previous study from India has also unveiled that women's education, wealth status, and region of residence are significant predictors of ANC utilization (50). Another study from India also reported that women from Muslim religion, scheduled castes, scheduled tribes, and other backward classes are less likely to use maternal health services (51). Similarly, other researchers have also established a positive association between household wealth (52) and education level (53) on ANC service utilization.

Fifth, exposure to health care messages has a significant influence on the utilization of ANC services (45). In our study, this finding was not significant. It is important that health care messages are tailored and targeted based on the existing situation, norms, beliefs, and practices. VHSND meetings are an excellent medium to extend such messages. However, without the empowerment of women, knowledge extension might be only half a battle won. Higher autonomy is reflected in the ability of women to decide whether to attend ANC clinics and makes it more likely for women to utilize the services (20, 54). Our study showed that having decision-making power or autonomy to visit relatives might be associated with high first trimester registration and utilization of ANC services. Other areas of decision making, such as autonomy to make household purchases, have a positive effect on uptake of tetanus injections and deworming tablets.

The currently pregnant adolescent women have shown the better utilization of all of the selected indicators of ANC than their adult counterparts. However, this does not mean that adolescent pregnancy should be promoted as early pregnancy (teenage pregnancy). Creating awareness about crucial aspects of ANC services should be stressed to address the issue of underutilization of ANC services among pregnant women. However, it is important to recognize that the aversion of teenage pregnancies is essential to reduce the risks related to maternal and child health issues. Improving the use of maternal health services requires a multipronged approach, including systems strengthening and contributions from women's groups, government sector, health professionals, and society. The study suggests that utilization of full ANC services, starting from the first trimester itself, for adolescent pregnant women, is an urgent need in the present context. Also, the finding of the present study that utilization of ANC services is better among adolescents is contrary to the general belief of researchers and indicates the need for further research to understand the factors leading to the higher rates of ANC utilization among adolescents.

## Data Availability Statement

All datasets generated for this study are included in the article/supplementary material.

## Ethics Statement

Ethical approval was taken from the Institutional Ethics Committee of All India Institute of Medical Sciences (AIIMS) Bihar, Chhattisgarh, and Odisha. The impact evaluation has been registered with the Registry for International Development Impact Evaluations (RIDIE-STUDY-ID-58261b2f46876) and Indian Council of Medical Research (ICMR) National Clinical Trials Registry of India (CTRI/2016/11/007482). Verbal consent was obtained from interviewed pregnant women. All India Institute of Medical Sciences Bihar, Chhattisgarh, and Odisha played a crucial role in data collection, data compilation, hiring and imparting intensive training to the field investigators, with technical support of International Institute for Population Sciences and UNICEF.

## Author Contributions

PF did conceptualization, data curation, formal analysis, and methodology. VS conceptualized, supervised, and validated and wrote, reviewed, and edited the original draft. RP and SB did conceptualization, investigation, project administration, and supervision. SS, AP, BM, and NK did acquisition, investigation, and administration. RG was involved in the investigation and administration of the study. NA, VB, and MR did conceptualization, investigation, and supervision of the study. AD did conceptualization and writing, review, and editing. ZM critically revised the manuscript. AS and KD did data curation, formal analysis, methodology and writing, and editing. NS did writing of the original draft and editing. All authors read and approved the submitted version.

### Conflict of Interest

The authors declare that the research was conducted in the absence of any commercial or financial relationships that could be construed as a potential conflict of interest.

## References

[1] Office of the Registrar General and Census Commissioner (2011): Census of India 2011 C-Series/ C-14 Five Year Age Group Data by Residence and Sex. New Delhi: Ministry of Home Affairs, Government of India.

[2] Chandra-Mouli V Camacho AV Michaud PA. WHO guidelines on preventing early pregnancy and poor reproductive outcomes among adolescents in developing countries. J Adolesc Health. (2013) 52:517. 10.1016/j.jadohealth.2013.03.002 23608717

[3] WHO Early Marriages, Adolescent and Young Pregnancies. World Health Organization (2011). Available online at: http://apps.who.int/gb/ebwha/pdf_files/EB130/B130_12-en.pdf (accessed January 9, 2019).

[4] IIPS and ICF National Family Health Survey (NFHS-4) 2015-16: India. (2017). Available online at: http://rchiips.org/NFHS/NFHS-4Report.shtml (accessed January 5, 2019).

[5] Kamal SMM Factors affecting utilization of skilled maternity care services among married adolescent in Bangladesh. Asian Pop Stud. (2009) 5:153 10.1080/17441730902992075

[6] Bhandari SD Joshi S Perception and perceived experiences about prevention and consequences of teenage pregnancy and childbirth among teenage mothers: a qualitative study. J Adv Acad Res. (2017) 3:164–72. 10.3126/jaar.v3i1.16625

[7] Althabe F Moore JL Gibbons L Berrueta M Goudar SS Chomba E. Adverse maternal and perinatal outcomes in adolescent pregnancies: the Global Network's Maternal Newborn Health Registry study. Reprod Health. (2015) 12:S8. 10.1186/1742-4755-12-S2-S8 26063350 PMC4464033

[8] Fall CHD Sachdev HS Osmond C Restrepo-Mendez MC Victora C Martorell R. Association between maternal age at childbirth and child and adult outcomes in the offspring: a prospective study in five low-income and middle-income countries (COHORTS collaboration). Lancet Glob Health. (2015) 3:e366–77. 10.1016/S2214-109X(15)00038-8 25999096 PMC4547329

[9] Pradeep TS Kulkarni P Murthy MN Adolescent mothers: determinants and dimensions. Int J Community Med Public Health. (2017) 4:1104 10.18203/2394-6040.ijcmph20171332

[10] McCall SJ Bhattacharya S Okpo E Macfarlane GJ. Evaluating the social determinants of teenage pregnancy: a temporal analysis using a UK obstetric database from 1950 to 2010. J Epidemiol Community Health. (2015) 69:49–54. 10.1136/jech-2014-204214 25227769

[11] Yakubu I Salisu WJ. Determinants of adolescent pregnancy in sub-Saharan Africa: a systematic review. Reprod Health. (2018) 15:15. 10.1186/s12978-018-0460-4 29374479 PMC5787272

[12] International Council for Research on Women New Insights on Preventing Child Marriage: A Global Analysis of Factors and Programs. (2007). Available online at: http://lastradainternational.org/lsidocs/icrw_child_marriage_0607.pdf (accessed October 22, 2018).

[13] UNICEF Progress for Children 2007 - Child Marriage. Unicef.org (2007). Available online at: https://www.unicef.org/progressforchildren/2007n6/index_41848.htm (accessed November 29, 2018).

[14] Narayana R Siddalingappa H Mishra B Assessment of utilization of antenatal care services by mothers attending immunization sessions at a primary health centre in Mysore district, Karnataka, India. Int J Community Med Public Health. (2016) 3:2561–65. 10.18203/2394-6040.ijcmph20163072

[15] WHO WHO Guidelines on Preventing Early Pregnancy and Poor Reproductive Outcome Among Adolescents in Developing Countries. WHO (2011). Available online at: https://www.who.int/immunization/hpv/target/preventing_early_pregnancy_and_poor_reproductive_outcomes_who_2006.pdf?ua=1 (accessed January 19, 2019).

[16] WHO WHO Recommendations on Adolescent Health: Guidelines approved by the WHO Guidelines Review Committee. (2017). Available online at: https://apps.who.int/iris/bitstream/handle/10665/259628/WHO-MCA-17.09-eng.pdf;jsessionid = 7171037D60D6FB790C44868C8914B933?sequence = 1 (accessed October 11, 2018).

[17] Bwalya BC Sitali D Baboo KS Zulu JM. Experiences of antenatal care among pregnant adolescents at Kanyama and Matero clinics in Lusaka district, Zambia. Reprod Health. (2018) 15:124. 10.1186/s12978-018-0565-9 29986756 PMC6038345

[18] Paudel YR Jha T Mehata S. Timing of First antenatal care (ANC) and inequalities in early initiation of ANC in Nepal. Front Public Health. (2017) 5:242. 10.3389/fpubh.2017.00242 28955707 PMC5600995

[19] WHO WHO Recommendations on Antenatal Care for a Positive Pregnancy Experience. (2016). Available online at: http://apps.who.int/iris/bitstream/10665/250796/1/9789241549912-eng.pd (accessed November 6, 2018).

[20] Shahabudin ASM Delvaux T Abouchadi S Sarker M De Brouwere V Utilization of maternal health services among adolescent women in Bangladesh: a scoping review of the literature. Trop Med Int Health. (2015) 20:822–29. 10.1111/tmi.12503 25757880

[21] Ministry of Health and Family Welfare Guidelines for Antenatal Care and Skilled Attendance at Birth by ANMs, LHVs/SNs. New Delhi: Government of India (2010). Available online at: https://www.nhp.gov.in/sites/default/files/anm_guidelines.pdf (accessed November 18, 2018).

[22] Mekonnen Y Mekonnen A. Factors influencing the use of maternal healthcare services in Ethiopia. J Health Popul Nutr. (2003) 21:374–82. 15038593

[23] Sahito A Fatmi Z. Inequities in antenatal care, and individual and environmental determinants of utilization at national and sub-national level in Pakistan: a multilevel analysis. Int J Health Policy Manag. (2018) 7:699–710. 10.15171/ijhpm.2017.148 30078290 PMC6077283

[24] IIPS and Macro International National Family Health Survey (NFHS-3) 2005–06: India. (2007). Available online at: http://rchiips.org/NFHS/nfhs3.shtml# (accessed January 5, 2019).

[25] Moller AB Petzold M Chou D Say L. Early antenatal care visit: a systematic analysis of regional and global levels and trends of coverage from 1990 to 2013. Lancet Glob Health. (2017) 5:e977–83. 10.1016/S2214-109X(17)30325-X 28911763 PMC5603717

[26] Ali N Sultana M Sheikh N Akram R Mahumud RA Asaduzzaman M. Predictors of optimal antenatal care service utilization among adolescents and adult women in Bangladesh. Health Serv Res Manag Epidemiol. (2018) 5:2333392818781729. 10.1177/2333392818781729 30083573 PMC6069020

[27] Boamah SA Amoyaw J Luginaah I. Explaining the gap in antenatal care service utilization between younger and older mothers in Ghana. J BiosocSci. (2016) 48:342–57. 10.1017/S0021932015000218 26160032

[28] Kisuule I Kaye DD Najjuka F Ssematimba SK Arinda A Nakitende G Timing and reasons for coming late for the first antenatal care visits by pregnant women at Mulango, Kampala Uganda. BMC Pregnancy Childbirth. (2013) 13: 11–7. 10.1186/1471-2393-13-121 23706142 PMC3665546

[29] Bearinger LH Sieving RE Ferguson J Sharma V. Global perspectives on the sexual and reproductive health of adolescents: patterns, prevention, and potential. Lancet. (2007) 369:1220–31. 10.1016/S0140-6736(07)60367-5 17416266

[30] Ziblim SD Yidana A Mohammed AR Determinants of antenatal care utilization among adolescent mothers in the Yendi municipality of northern region, Ghana. Ghana J Geography (2018) 10:78–97.

[31] Reserve Bank of India Handbook of Statistics on the Indian Economy 2018-19. Mumbai: Reserve Bank of India (2019). Available online at https://rbidocs.rbi.org.in/rdocs/Publications/PDFs/0HB2018-19A91A298806164470A2BCEF300A4FE334.PDF (accessed September 27, 2019).

[32] Clinical Trials Registry India (CTRI) Integrated Multisectoral Strategy to Improve Girls' and Women's Nutrition Before Conception, During Pregnancy and After Birth in India (Swabhimaan). Identifier CTRI/2016/11/007482. New Delhi: Indian Council of Medical Research (India) (2016). Available online at: http://ctri.nic.in/Clinicaltrials/showallp.php?mid1=16738&EncHid=&userName=Swabhimaan (accessed January 24, 2019).

[33] Reshmi RS Dinachandra K Bhanot A Unisa S Menon GT Agrawal N Context for layering women's nutrition interventions on a large scale poverty alleviation program: Evidence from three eastern Indian states. PLoS ONE. (2019) 14:e0210836 10.1371/journal.pone.0210836 30668595 PMC6342298

[34] Mumbare SS Rege R. Ante natal care services utilization, delivery practices and factors affecting them in tribal area of north Maharashtra. Indian J Community Med. (2011) 36:287–90. 10.4103/0970-0218.91331 22279259 PMC3263149

[35] Kakati R Barua K Borah M Factors associated with the utilization of antenatal care services in rural areas of Assam, India. Int J Community Med Public Health. (2016) 3:2799–805. 10.18203/2394-6040.ijcmph20163364

[36] Adhikari T Sahu D Nair S Saha KB Sharma RK Pandey A. Factors associated with utilization of antenatal care services among tribal women: a study of selected States. Indian J Med Res. (2016) 144:58–66. 10.4103/0971-5916.193284 27834327 PMC5116899

[37] Gross K Alba S Glass TR Schellenberg JA Obrist B. Timing of antenatal care for adolescent and adult pregnant women in south-eastern Tanzania. BMC Pregnancy Childbirth. (2012) 12:16. 10.1186/1471-2393-12-16 22436344 PMC3384460

[38] Banke-Thomas OE Banke-Thomas AO Ameh CA. Factors influencing utilisation of maternal health services by adolescent mothers in low-and middle-income countries: a systematic review. BMC Pregnancy Childbirth. (2017) 17:65. 10.1186/s12884-017-1246-3 28209120 PMC5314631

[39] Kerber KJ de Graft-Johnson JE Bhutta ZA Okonq P Starrs A Lawn JE. Continuum of care for maternal, newborn, and child health: from slogan to service delivery. Lancet. (2007) 370:1358–69. 10.1016/S0140-6736(07)61578-5 17933651

[40] Oringanje C Meremikwu MM Eko H Esu E Meremikwu A Ehiri JE. Interventions for preventing unintended pregnancies among adolescents. Cochrane Database Syst Rev. (2016) 2:CD005215. 10.1002/14651858.CD005215.pub3 26839116 PMC8730506

[41] WHO Pregnant Adolescents: Delivering on Global Promises of Hope. Geneva: WHO (2006). Available online at: https://apps.who.int/iris/bitstream/handle/10665/43368/9241593784_eng.pdf?sequence=1&isAllowed=y (accessed December 9, 2018).

[42] Magadi MA Agwanda AO Obare FO. A comparative analysis of the use of maternal health services between teenagers and older mothers in sub-Saharan Africa: evidence from Demographic and Health Surveys (DHS). Soc Sci Med. (2007) 64:1311–25. 10.1016/j.socscimed.2006.11.004 17174017

[43] Reynolds HW Wong EL Tucker H. Adolescents' use of maternal and child health services in developing countries. Int Fam Plan Perspect. (2006) 32:6–16. 10.1363/3200606 16723297

[44] Worku EB Woldesenbet SA. Factors that influence teenage antenatal care utilization in John TaoloGaetsewe (JTG) district of Northern Cape Province, South Africa: underscoring the need for tackling social determinants of health. Int J MCH AIDS. (2016) 5:134–45. 28058200 PMC5187645

[45] Ndambuki SM Bidemi YO Aimakhu CO Factors influencing utilization of antenatal care services among teenage mothers in Malindi Sub-County Kenya- A cross-sectional study. Sci J Public Health. (2017) 5:61–7. 10.11648/j.sjph.20170502.12

[46] Alsahafi NA Bukhari AA Abokashabah SA Al-zahidy ZA Alshareef EA Bajouh OS Obstacles affecting antenatal care attendance: results from a cross sectional study in Jeddah, Saudi Arabia. E Cronicon Gynaecol. (2016) 2.3:213–9.

[47] Adewunmi A Rabiu K Tayo A Gestational age at antenatal booking in Lagos, South-West Nigeria. Int J Gynecol Obstet. (2008) 12:1–4. 10.5580/891

[48] Ministry of Health and Family Welfare Rashtriya Kishor Swasthya Karyakram. Operational Framework- Translating Strategy Into Programmes. New Delhi: Adolescent Health Division, Government of India (2014). Available online at: http://nhm.gov.in/images/pdf/programmes/RKSK/RKSK_Operational_Framework.pdf (accessed November 6, 2018).

[49] Brahmapurkar KP Poor maternal health care in rural districts: an attempt to identify high priority districts of Bihar, India. Natl J Community Med. (2017) 8:79–83.

[50] Singh PK Rai RK Alagarajan M Singh L. Determinants of maternity care services utilization among married adolescents in rural India. PLoS ONE. (2012) 7:e31666. 10.1371/journal.pone.0031666 22355386 PMC3280328

[51] Singh A Kumar A Pranjali P. Utilization of maternal healthcare among adolescent mothers in urban India: evidence from DLHS-3. Peer J. (2014) 2:e592. 10.7717/peerj.592 25392750 PMC4226640

[52] Singh PK Kumar C Rai RK Singh L. Factors associated with maternal healthcare services utilization in nine high focus states in India: a multilevel analysis based on 14385 communities in 292 districts. Healthy Policy Plan. (2014) 29:542–59. 10.1093/heapol/czt039 23783832

[53] John AE Nilima Binu VS Unnikrishnan B. Determinants of antenatal care utilization in India: a spatial evaluation of evidence for public health reforms. Public Health. (2019) 166:57–64. 10.1016/j.puhe.2018.09.030 30453146

[54] Chaibva CN Roos JH Ehlers VJ. Adolescent mothers' non-utilisation of antenatal care services in Bulawayo, Zimbabwe. Curationis. (2009) 32:14–21. 10.4102/curationis.v32i3.1219 20225740

